# Enhanced pathogen identification among patients with clinically suspected meningitis

**DOI:** 10.4102/sajid.v39i1.688

**Published:** 2024-12-10

**Authors:** Malefu Moleleki, Pieter Nel, Siphiwe R. Matukane, Stephanie Cloete, Zayaan Abrahams, Nicole Wolter, Andrew C. Whitelaw

**Affiliations:** 1 Division of Medical Microbiology, Department of Pathology, Faculty of Medicine and Health Sciences, Stellenbosch University, Cape Town, South Africa; 2 Department of Clinical Microbiology and Infectious Diseases, School of Pathology, Faculty of Health Sciences, University of the Witwatersrand, Johannesburg, South Africa; 3 Division of Medical Microbiology, Tygerberg Hospital, National Health Laboratory Service, Cape Town, South Africa; 4 Centre for Respiratory Diseases and Meningitis, National Institute for Communicable Diseases of the National Health Laboratory Service, Johannesburg, South Africa

**Keywords:** meningitis, PCR, multi-pathogen testing, molecular testing, aetiology, syndromic

## Abstract

**Background:**

Delayed or incorrect treatment of meningitis may result in adverse patient outcomes. However, laboratory testing in resource-limited settings is often limited to conventional diagnostic methods. We explored the utility of syndromic molecular assays for diagnosis.

**Objectives:**

We tested cerebrospinal fluid (CSF) specimens collected from patients with clinically suspected meningitis submitted to a tertiary hospital laboratory in January 2021 – May 2021. Primary microbiological analysis (culture, Gram stain and cytochemical analysis) was performed as part of routine testing.

**Method:**

Residual CSF specimens were tested using a bacterial triplex real-time polymerase chain reaction (PCR) assay and a syndromic multi-pathogen real-time PCR assay for the detection of up to 18 bacterial and viral pathogens. Pathogen detection was compared between conventional and molecular assays.

**Results:**

A potential pathogen was detected in 6% (12/188) and 47% (89/188) of specimens on the triplex and the multi-pathogen assay, respectively. Epstein-Barr virus (49/188; 26%), human herpes virus 7 (22/188; 12%), herpes simplex virus 1 (13/188; 7%) and *Streptococcus pneumoniae* (10/188; 5%) were the leading pathogens detected on the syndromic multi-pathogen PCR. Further, using the multi-pathogen PCR assay, a potential pathogen was detected in 44% (73/166) of the specimens which were negative following routine testing. Overall, combining routine testing and molecular platforms significantly improved pathogen detection (*p* < 0.001); a potential pathogen was identified in 51% (95/188) of the specimens tested, compared to 12% (22/188) using routine methods alone.

**Conclusion:**

The use of molecular tests improved pathogen detection by 39% when paired with routine methods.

**Contribution:**

Multi-pathogen molecular testing is useful for rapidly diagnosing meningitis cases.

## Introduction

Meningitis remains a significant cause of morbidity and mortality in early childhood and adolescence, with the highest incidence occurring in low- and middle-income countries.^1^ The greatest burden is in sub-Saharan Africa, accounting for approximately half of the estimated 2.52 million cases of meningitis which occurred globally in 2019.^1^ Untreated bacterial meningitis is fatal in approximately 50% of cases and delaying treatment may result in long-term neuropsychological sequelae in 20% – 25% of survivors.^2^

The predominant aetiological cause of meningitis varies by age, with Group B streptococcus (GBS): *Escherichia coli, Klebsiella pneumoniae* and *Listeria monocytogenes* commonly identified in early childhood.^1^ Prior to vaccine introduction, *Streptococcus pneumoniae, Neisseria meningitidis* and *Haemophilus influenzae* type b (Hib) were the most common causes of meningitis in older children and adults, globally.^2^ Viral aetiologies are more common in childhood meningitis but rarely result in fatal disease.^3^ Common viral aetiologies include coxsackieviruses and echoviruses,^4^ with a wide variety of less common aetiologies such as adenovirus (AdV), mumps virus (MV) and herpesviruses.^5^ In South Africa, because of the high burden of HIV infections, *Cryptococcus neoformans* and *Mycobacterium tuberculosis* also play an important role. Terwin et al. reported a 16% and 37% prevalence of these pathogens, respectively, among adult meningitis cases presenting to a district hospital in South Africa.^6^

Current guidelines for the management of acute meningitis in South Africa recommend empiric administration of antibiotics prior to hospitalisation and laboratory testing.^7^ However, it is challenging to reliably differentiate between viral and bacterial meningitis based on clinical presentation alone. This may contribute to > 80% of paediatric patients with viral meningitis receiving antibiotics inappropriately.^7,8^ The ability to rapidly exclude bacterial meningitis or confirm a viral pathogen can result in reduced antibiotic consumption with beneficial downstream impacts such as the prevention of antimicrobial resistance, unnecessary hospitalisation costs and hospital-acquired infections. Conversely (and less commonly), patients with an atypical presentation of acute bacterial meningitis may not receive antibiotics, and rapid detection of a bacterial pathogen may result in earlier and more targeted administration of antibiotic therapy.

Laboratory testing in resource-limited settings is often limited to conventional diagnostic methods such as microscopy, which has low sensitivity, and culture which is limited to bacteria and fungi and depends on the organism’s viability.^7^ Nucleic acid amplification tests are a gold standard for viral testing and improve the sensitivity and turnaround time of bacterial detection compared to culture.^9^ Syndromic, multi-pathogen real-time polymerase chain reaction (PCR) assays can simultaneously detect a wide range of pathogens from a single specimen. These platforms allow for a rapid identification of disease aetiology, particularly in outbreak situations.^10,11,12,13,14^

We explored the clinical utility of syndromic molecular tests for the detection of meningitis-causing pathogens in our facility. This included a triplex real-time PCR assay for the detection of *S. pneumoniae, H. influenzae* and *N. meningitidis* and a syndromic real-time PCR assay for the detection of up to 18 pathogens.

## Research methods and design

### Sampling

We collected cerebrospinal fluid (CSF) specimens submitted as part of routine patient investigation to the National Health Laboratory Service pathology laboratories at Tygerberg Hospital, in the Western Cape province, South Africa, between January 2021 and May 2021. Patients of all ages with a presumed clinical suspicion of meningitis (based on the submission of CSF for microbiological analysis) were eligible. Primary microbiological analysis (culture, Gram stain and cytochemical analysis) was carried out as part of routine testing. Residual CSF specimens were stored at –80 °C until further testing. Our inclusion criteria for this study were as follows: adequate volume of residual CSF (≥ 200 µL) and an abnormal cell count (defined as a total leucocyte count of ≥ 5 cells/mm^3^).^7^ Demographic and clinical information was extracted from the laboratory information system.

**FIGURE 1:**
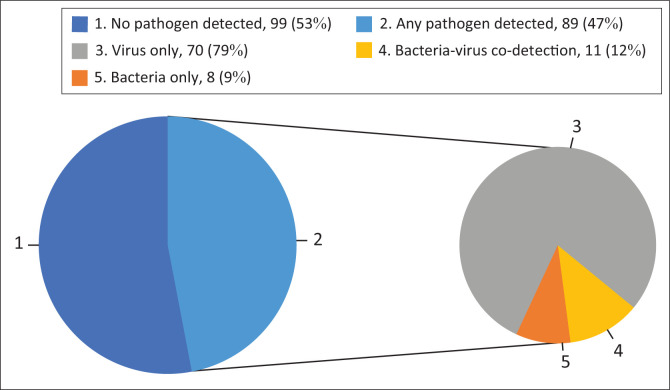
Pathogen detection in cerebrospinal fluid from patients with suspected meningitis tested using the multi-pathogen PCR platforms (*N* = 188).

Cerebrospinal fluid specimens were tested as described in Online Appendix 1, Figure 1-A1. For the current study, 371 CSF specimens were tested using an in-house triplex real-time PCR assay for the detection of historically common causes of meningitis. A random selection of 188 (51%) of these specimens were further tested using a commercial multi-pathogen real-time PCR platform for the detection of six bacterial species and 12 viruses.

### Triplex real-time polymerase chain reaction

Total nucleic acid (TNA) was extracted from 200 µL CSF using the Maxwell RSC automated extractor and the Maxwell RSC Viral Total Nucleic Acid purification kit (Promega, Wisconsin, United States) according to the manufacturer’s instructions and stored at –20 °C until further use. A real-time PCR assay targeting *S. pneumoniae, H. influenzae* and *N. meningitidis* was performed as previously described.^15^ A positive result was recorded if amplification occurred with cycle threshold value (Ct) < 40. To monitor nucleic acid extraction and assess PCR inhibition, an additional real-time PCR assay for the detection of a human gene, *RNaseP*, was performed as previously described by Carvalho et al.^16^ A positive result was recorded if amplification occurred with Ct < 35.

### Multi-pathogen real-time polymerase chain reaction

Total nucleic acid from a random subset of the specimens, selected using a random number generator, was tested using three commercial real-time PCR panels (Seegene Inc, Seoul, Republic of Korea) according to the manufacturer’s instructions. The Allplex Meningitis-B kit was used for the detection of *E. coli* serotype K1, GBS, *H. influenzae, S. pneumoniae, N. meningitidis* and *L. monocytogenes.* The Allplex Meningitis-V1 and V2 kits were used for the detection of cytomegalovirus (CMV), Epstein-Barr virus (EBV), herpes simplex virus type 1 (HSV1), human parechovirus (HPeV), herpes simplex virus type 2 (HSV2), human herpes viruses 6 and 7 (HHV6, HHV7), varicella-zoster virus (VZV), AdV, enterovirus (HEV), MV and parvovirus B19 (B19V). The assays were performed using the CFX96 real-time instrument (Bio-Rad Laboratories Inc., Hercules, CA, United States). A positive result was recorded if amplification occurred with Ct ≤ 45.

### Statistical analysis

Data analysis was performed using Stata 14 (Stata Corporation, College Station, TX, United States). For continuous data, the results were summarised into mean and standard deviation for symmetric variables and median and interquartile range for asymmetric variables. Categorical data were summarised into frequencies and percentages. Chi-squared or the Fisher’s exact tests were used, where appropriate, to determine associations between categorical variables, with *p* < 0.05 considered statistically significant. Concordance between the triplex PCR and the syndromic multi-pathogen platforms for the detection of *S. pneumoniae, H. influenzae* and *N. meningitidis* was calculated using overall percent agreement (OPA).

### Ethical considerations

Ethical approval to conduct the study was obtained from the Health Research Ethics Committee (HREC) of a university in the Western Cape (N20/05/061). Permission to use patient information was obtained from the Western Cape Department of Health. Patient data were handled confidentially and no identifying information was captured. A waiver of consent was obtained from the institutional HREC as testing was carried out on left-over specimens following routine microbiological analysis and at no risk to the human participants.

## Results

From January 2021 through May 2021, 371 CSF specimens were collected and tested using the triplex PCR while 188 (51%) were tested using the commercial syndromic PCR panels (Table 1). The median age of the patients tested was 26 years (interquartile range 1–38 years); 33% (123/371) were aged < 5 years and the majority of these were aged < 1 year (84/123; 68%).

**TABLE 1:** Demographic characteristics of the patients with suspected meningitis tested using molecular methods.

Characteristic	In-house triplex assay (*N* = 371)				Syndromic multi-pathogen PCR† (*N* = 188)			
	*n*	%	Median age	IQR	*n*	%	Median age	IQR
**Gender**								
Female	178	48	-	-	79	42	-	-
Age (years)	-	-	26	1–38	-	-	27	1–38
**Age group (years)**								
< 1	84	23	-	-	44	23	-	-
1–4	39	11	-	-	22	12	-	-
5–15	15	4	-	-	6	3	-	-
> 15	233	63	-	-	116	62	-	-
**CSF white blood count (cells/mm^3^)**								
< 10	130	35	-	-	64	34	-	-
10–100	186	50	-	-	89	47	-	-
> 100	55	15	-	-	35	19	-	-

CSF, cerebrospinal fluid; PCR, polymerase chain reaction; IQR, interquartile range.

† , The 188 samples tested using the syndromic multi-pathogen PCR represent a subset of the 371 samples tested using the triplex assay.

### Pathogen detection

Of the 371 CSF specimens tested using the triplex PCR, 28 (8%) tested positive. *Streptococcus pneumoniae* was the most common pathogen detected (23/371; 6%) followed by *N. meningitidis* (5/371; 1%), while *H. influenzae* was not detected. Of the CSF specimens testing PCR-positive for *S. pneumoniae* or *N. meningitidis*, culture identified these pathogens in 9/23 (39%) and 0/5 (0%) specimens respectively. Conversely, there were no culture-positive samples which were negative on the PCR for the three targets included. Overall, a pathogen remained undetected in 343/371 (92%) specimens following testing on the triplex assay.

Among the 188 CSF specimens tested using the syndromic panels, a pathogen had been detected in 12 (6%) using the triplex PCR with 99% OPA in the detection of *N. meningitidis* and *H. influenzae* compared to 97% OPA for *S. pneumoniae* (Online Appendix 1, Table 1-A1). Using the syndromic multi-pathogen assay, a pathogen was identified in 89/188 (47%) specimens: any bacteria in 19 (10%) and any virus in 81 (43%). A pathogen was not detected in 99/188 (53%) specimens following testing on the syndromic platform. Bacterial-only infections were identified in 8/188 (4%), while virus-only infections were identified in 70/188 (37%) specimens (Figure 1). Co-detections were identified in 36/188 (19%) specimens, of which bacterial–viral co-detections were identified in 11/188 (6%). Among the leading pathogens with a prevalence of ≥ 4%, EBV was commonly detected as a single pathogen (32/49; 65%), while HSV2 (8/8; 100%), HSV1 (12/13; 92%), HHV7 (17/22; 77%), HEV (4/6; 67%), *S. pneumoniae* (6/9; 67%) and *L. monocytogenes* (5/8; 63%), were commonly detected in combination with at least one other pathogen (Online Appendix 1, Table 2-A1).

Table 2 shows the prevalence of bacterial and viral pathogens detected using the three syndromic multi-pathogen PCR panels and stratified by age. Among these, *S. pneumoniae* (10/188; 5%) and *L. monocytogenes* (7/188; 4%) were the leading bacterial pathogens. *Listeria monocytogenes* was not detected using routine culture, while the culture-positivity rate for *S. pneumoniae* was 4% (7/188). Among the viral pathogens detected, EBV (49/188; 26%), HHV7 (22/188; 12%), HSV1 (13/188; 7%), HSV2 (8/198; 4%) and HEV (6/198; 3%) were the most common. Comparing pathogen prevalence by age, there was no significant difference in the prevalence of specific bacterial pathogens between patients aged < 5 years and those aged ≥ 5 years. On the other hand, any pathogen and any virus detected was significantly higher in those ≥ 5 year than in the younger patients. Among the viral pathogens, HHV6 (*p* = 0.042) and HHV7 (*p* = 0.017) were more commonly detected in children aged < 5 years, whereas EBV (*p* < 0.001) was more commonly detected among individuals aged ≥ 5 years.

**TABLE 2:** Pathogens detected using the multi-pathogen meningitis panels in cerebrospinal fluid collected from patients with a clinical suspicion of meningitis.

Pathogen detected	Prevalence of organism						
	All patients (*N* = 188)		Age < 5 years (*N* = 66)		Age ≥ 5 years (*N* = 122)		*p*
	*n*	%	*n*	%	*n*	%	
**Any pathogen**	89	47	23	35	66	54	0.014*
**Any bacteria**	19	10	5	8	14	11	0.610
*Streptococcus pneumoniae*	10	5	1	2	9	7	0.169
*Listeria monocytogenes*	7	4	3	5	4	3	0.698
*Haemophilus influenzae*	2	1	1	2	1	1	1.000
*Neisseria meningitidis*	1	1	0	0	1	1	ND
**Any viruses**	81	43	21	32	60	49	0.030*
EBV	49	26	4	6	45	37	< 0.001*
HHV7	22	12	13	20	9	7	0.017*
HSV1	13	7	3	5	10	8	0.548
HSV2	8	4	2	3	6	5	0.715
HEV	6	3	3	5	3	2	0.425
CMV	6	3	2	3	4	3	1.000
VZV	6	3	1	2	5	4	0.667
AdV	4	2	1	2	3	2	1.000
HHV6	3	2	3	5	0	0	0.042*
MV	3	2	1	2	2	2	1.000
B19V	1	1	0	0	1	1	ND

Note: The *p*-value for the Chi-squared test compared < 5 years to ≥ 5 years.

AdV, adenovirus; B19V, parvovirus B19; CMV, Cytomegalovirus; EBV, Epstein-Barr virus; HEV, enterovirus; HHV6, human herpes virus 6; HHV7, human herpes virus 7; HPeV, human parechovirus; HSV1, herpes simplex virus type 1; HSV2, herpes simplex virus type 2; MV, mumps virus; ND, not done; VZV, varicella-zoster virus.

* , *p* < 0.05 was considered statistically significant.

### Comparing routine testing with polymerase chain reaction for pathogen detection

We compared the results of routine microbiological testing and the syndromic multi-pathogen molecular assays on 188 specimens with results available for both platforms. A pathogen was detected in 22 (12%) specimens by routine testing, with *C. neoforman*s (11/188; 6%) and *S. pneumoniae* (7/188; 4%) being the most common. Routine culture further identified pathogens which were not included as targets on the multi-pathogen PCR assays in 4/188 (2%) specimens. These pathogens included *Acinetobacter baumannii* and coagulase negative staphylococci. Culture identified *S. pneumoniae* in 7/10 (70%) of the specimens positive on multi-pathogen PCR but did not detect *L. monocytogenes, H. influenzae* and *N. meningitidis*, all of which were identified using PCR. Among the 166 specimens that were negative by routine culture, the multi-pathogen PCR assay yielded a pathogen in 73/166 (44%) specimens, of which 6/166 (4%) were bacteria only, 61/166 (37%) were viruses only and bacterial–viral co-detections were identified in 6/166 (4%). Overall, when combining routine testing and the syndromic multi-pathogen platforms, a pathogen was identified in 51% (95/188) of the specimens tested, compared to 12% (22/188) using routine methods alone.

## Conclusion and discussion

We used PCR-based assays for the identification of bacterial and viral pathogens causing meningitis at a tertiary hospital in the Western Cape, South Africa, from January 2021 to May 2021. We identified at least one pathogen in 47% of specimens on a multi-pathogen real-time PCR platform able to detect up to six bacterial pathogens and 12 viruses. Compared to routine microbiological testing which identified a pathogen in 12% of the specimens, we were able to improve our detection rate by 35% using the multi-pathogen PCR platform.

Of the specimens which had remained negative following routine testing, the multi-pathogen PCR assays identified a pathogen in 44% of the samples. Among these specimens, bacteria accounted for 8% of the pathogens detected using PCR. The higher detection rate of bacteria using PCR can be attributed to the platform not requiring a viable organism for detection, unlike culture, but can detect low amounts of nucleic acids from any targeted organism. Meningitis is a medical emergency and thus clinicians are required to prioritise empirical antibiotic therapy particularly if there are delays in lumbar puncture procedures.^7^ Initiation of antibiotic therapy prior to CSF collection may have contributed to the 8% culture-negative but bacterial PCR-positive results. The low positivity of culture compared to bacterial PCR has been reported previously.^17,18^ In our study, while culture and PCR were comparable for *S. pneumoniae* detection, PCR positivity was higher for the detection of *L. monocytogenes* (4%) which was not detected using culture. The same findings were notable for the rest of the bacterial pathogens detected using the multi-pathogen platforms though at a low positivity rate.

In our study, *S. pneumoniae* (5%) was the leading bacterial pathogen detected using the multi-pathogen PCR platform, in line with what has been previously reported in South Africa and elsewhere in the post- pneumococcal conjugate vaccine (PCV) era.^17,19,20^ Globally and in our setting, invasive pneumococcal disease with vaccine serotypes decreased in the past two decades since the introduction of the PCV into childhood immunisation programmes.^21,22,23^ However, in our setting, pneumococcus remains the leading cause of acute bacterial meningitis despite the introduction of the PCV7 and later PCV13 into the childhood immunisation programme.^20^ Britz et al., reported a 1.1% *S. pneumoniae* prevalence among 110 885 CSF specimens collected between 2009 and 2012 in patients with a clinical suspicion of meningitis in the Gauteng Province, South Africa.^20^ In their study, *C. neoformans* (6.7%) and *M. tuberculosis* (2.6%) were the leading pathogens, not unexpected with the high HIV prevalence in this province.^20^ In our study, *C. neoformans* was detected at 6% based on routine culture; however, *M. tuberculosis* testing was performed only on request by clinicians and so was not included in the analysis. Both these pathogens, clinically important in our high HIV prevalence setting, were not included as targets for the multi-pathogen PCR, which is a limitation of these commercial kits.

We identified *L. monocytogenes* (4%) as the second leading bacterial pathogen based on the multi-pathogen PCR, with comparable prevalence among patients aged < 5 years and older children and adults. This pathogen commonly causes meningitis in young infants, the elderly and among the immunocompromised patient population.^24^ In their systematic review and meta-analysis, De Noordhout et al. estimated that in 2010, globally, neurolisteriosis resulted in 1.24 disability-adjusted life-years (DALYs) per 100 000 people.^24^ However, in South Africa, Britz et al. identified only 16 cases of *L. monocytogenes* meningitis among 110 885 CSF specimens tested between 2009 and 2012 in the Gauteng Province.^20^ The low prevalence in their study may be because of microbiological analysis differences; in their study, Britz et al. defined acute bacterial meningitis cases based on a positive culture, and they excluded results obtained using molecular tests. In our study, *L. monocytogenes* was only detected using the multi-pathogen PCR test and none detected using culture. *Listeria monocytogenes* is a common foodborne pathogen which commonly causes outbreaks of listeriosis worldwide, with the largest outbreak in history occurring in South Africa between 2017 and 2018.^25^ During this outbreak period, a case was defined on the basis of a positive culture or a PCR-based test, highlighting the importance of both methods for enhanced pathogen detection.

In our study, viruses were detected in 43% of specimens with some notable differences in frequencies between the age groups. Epstein-Barr Virus (26%), HHV7 (12%), HSV1 (7%), HSV2 (4%) and HEV (3%) were the leading viruses detected, with EBV more common in older children and adults and HHV6 and HHV7 more common in younger children. Human enteroviruses are common causes of aseptic meningitis across all age groups accounting for 30% – 75% of all cases with the variations in prevalence dependent on the season, age group and geographical region.^26,27^ Therefore, the 3% prevalence of HEV in our study is far below what has been reported elsewhere and is likely a result of a reduction in viral transmission because of the coronavirus disease 2019 (COVID-19) pandemic lockdowns during the study period as previously reported in South Africa.^28^ Further, HSV-1 is a recognised cause of viral encephalitis and its presence in CSF would typically prompt a clinical action. On the other hand, the detection of other herpesviruses, such as HHV6, HHV7 and EBV, in CSF might represent latent infection or asymptomatic viral reactivation, therefore detection may not indicate causality.^29^ These viruses can infect neurons or endothelial cells of blood vessels in the brain, causing latent infection that can be reactivated when the brain experiences stress resulting in an inflammatory response.^29^ Infections often occur in early childhood, and while the disease may be self-limiting in adults, it can be severe in infants and younger children with increased mortality in these age groups. Therefore, while they may have been detected as leading viruses in the study, careful interpretation is required as their DNA can be found in the CSF as a possible bystander to other infections of the central nervous system, such as tuberculosis, bacterial meningitis and herpes simplex encephalitis.^30^ It is also our finding that, with the exception of EBV, these viruses often occurred as co-pathogens with either bacteria or other viruses.

Limitations of our study included the absence of a confirmatory test where results between routine testing and the molecular assays were discordant. This may have resulted in an overestimation of the roles of some of the pathogens, such as *L. monocytogenes*, in disease. Further, to determine the clinical relevance of the pathogens, particularly the viruses including HHV6, HHV7 and EBV, additional laboratory and clinical information such as CSF protein and glucose analyses, HIV status, immunosuppression and co-morbidities would be useful. Future studies should include the collection and interrogation of these data to support laboratory results interpretation. Also, the targeted multi-pathogen PCR could not be customised to include pathogens of clinical importance in our setting, such as *M. tuberculosis* and *C. neoformans*. The exclusion of these pathogens, especially in our high HIV prevalence setting is particularly concerning as it limits the utility of the test for rapid diagnosis. However, for routine diagnosis, cryptococcal antigen tests and PCR-based molecular tests for *M. tuberculosis* detection are available in our facility. Lastly, the study period of 5 months, from January 2021 to May 2021, could have limited our ability to detect some pathogens because of the inherent seasonality of these pathogens. Future studies should be conducted over a longer period to accommodate pathogen seasonality and periodicity.

In conclusion, use of the multi-pathogen real-time PCR platforms enhanced pathogen detection by 44% among individuals with suspected meningitis among the specimens which were negative following routine testing. Further, combining molecular testing with routine microbiological testing identified a pathogen in 51% of the specimens tested. Rapid and comprehensive testing for common meningitis pathogens has the potential to contribute to patient management to improve outcomes.
